# A Novel Biocompatible Herbal Extract-Loaded Hydrogel for Acne Treatment and Repair

**DOI:** 10.1155/2021/5598291

**Published:** 2021-11-02

**Authors:** Ying-Yi Lin, Shu-Hsu Lu, Rong Gao, Chia-Hung Kuo, Wen-Hisn Chung, Wei-Chih Lien, Ching-Chou Wu, Yong Diao, Hui-Min David Wang

**Affiliations:** ^1^Graduate Institute of Biomedical Engineering, National Chung Hsing University, Taichung City 402, Taiwan; ^2^Division of Cardiology, Department of Internal Medicine, Kaohsiung Armed Forces General Hospital, Kaohsiung City, Taiwan; ^3^Deloitte Institute of Biology, Yangtze River Delta Research Institute, Tsinghua University, Beijing, China; ^4^Department of Seafood Science, National Kaohsiung University of Science and Technology, Kaohsiung City, Taiwan; ^5^Department of Plant Pathology, National Chung Hsing University, Taichung City 402, Taiwan; ^6^Department of Physical Medicine and Rehabilitation, National Cheng Kung University Hospital, College of Medicine, National Cheng Kung University, Tainan 704, Taiwan; ^7^Department of Physical Medicine and Rehabilitation, College of Medicine, National Cheng Kung University, Tainan 701, Taiwan; ^8^Ph.D. Program in Tissue Engineering and Regenerative Medicine, National Chung Hsing University, Taichung City 402, Taiwan; ^9^Department of Bio-Industrial Mechatronics Engineering, National Chung Hsing University, Taichung City 402, Taiwan; ^10^Innovation and Development Center of Sustainable Agriculture, National Chung Hsing University, Taichung City 402, Taiwan; ^11^School of Medicine, Huaqiao University, Quanzhou, Fujian Province 362021, China; ^12^Graduate Institute of Medicine, College of Medicine, Kaohsiung Medical University, Kaohsiung City, Taiwan; ^13^Department of Medical Laboratory Science and Biotechnology, China Medical University, Taichung City, Taiwan

## Abstract

A novel herbal extract-loaded gel containing several biofunctional extracts, including green tea, *Zingiber officinale* Rosc, *Phyllanthus emblica*, and salicylic acid, was developed for acne vulgaris. These natural raw materials were blended with suitable dosages of gelatin and carboxymethyl cellulose (CMC) to produce a biocompatible herbal gel. The physical chemistry properties of the hydrogel were determined by Fourier transform infrared spectroscopy (FTIR), thermal gravimetric analysis (TGA), rheometry, and scanning electron microscopy (SEM), and the hydrogel showed good mechanical and morphological characteristics. The herbal extract-loaded hydrogel mimicked extracellular matrix properties and showed good antioxidant and anti-inflammatory properties and various advantages, serving as a potential wound dressing material because of its high moisture retention ability, wound exudate absorption behavior, and biocompatibility. It exhibited moderate-high antioxidative and anti-inflammatory qualities that were important for dermis wound closure. The clinical trial results showed that most patients experienced moderate to high healing rates, and four of twenty-four individuals (16.67%) had recovery area ratios greater than 80%. This herbal extract-loaded hydrogel has effective ingredients and excellent mechanical properties as a bioactive dressing agent for acne treatment.

## 1. Introduction

Acne vulgaris is an unpleasant facial skin problem with a prevalence of 80% and affects social interactions, especially for teenagers [1]. The presence of acne can reduce self-esteem and self-confidence; therefore, an effective treatment is beneficial to quality of life for adolescents. Four major causes of acne include (1) high sebum production from sebaceous glands, (2) blockade of keratinization within pilosebaceous follicles, (3) rapid growth and proliferation of microorganisms such as *Propionibacterium acnes*, and (4) an immune inflammatory response and swelling fortification around pilosebaceous follicles [2].

Acne development is triggered by interactions between the sebostatic reaction associated with sebum deregulation and inflammatory reactions. Various complementary medicines, such as natural product extracts, plant oils, and antimicrobial peptides, have few side effects [2–4]. Previous studies showed that green tea polyphenols with anti-inflammatory and antibacterial characteristics heal acne infections and reduce sebum secretion in the dermis [5]. Ginger constitutes have antiseptic and anti-inflammatory properties and increase cellular migration [6–8]. *Phyllanthus emblica* fruit extract powder abated antioxidative stress injuries and suppressed inflammatory responses [9, 10]. The antimicrobial activities of *P. emblica*, green tea, and ginger have been investigated by a number of previous studies [11–13]; these active extracts showed antibacterial activity against gram-positive bacteria (*Staphylococcus aureus*) and gram-negative bacteria (*Escherichia coli* and *Pseudomonas aeruginosa*). Salicylic acid dissolved skin debris, which clogged pores and caused acne progression as a lipophilic agent, thus decreasing corneocyte cohesion and promoting desquamation, particularly of the hydrophobic upper layers in the stratum corneum [1]. Previous studies by this group showed that these bioactive components can be used to treat acne through biofunctions.

A biomedical hydrogel dressing fabricated from natural extracts was used as a scaffold to efficiently deliver active constituents for acne repair and to reconstruct skin tissue [14]. Gelatin is a denatured protein product from collagen that is present at high levels within the extracellular matrix and is usually used in the biomedical and pharmaceutical industries [15–17]. Gelatin is accessible, economical, nonpoisonous, and biocompatible and has weak antigenic properties and many peptide sequences, such as arginine glycine-aspartic acid, which increases cellular adhesion [18]. Carboxymethyl cellulose (CMC) is also a harmless biocompatible material used for wound repair [19]. CMC compensates for the poor cohesion of gelatin in a wound healing gel when used as a base polymer.

Herbal extract-loaded hydrogels enhance water retention, absorb exudate around the wound site, increase pliability and biocompatibility, and resemble extracellular matrix in a wound dressing material. In our previously published paper, we showed that our hydrogel was biocompatible and nontoxic to foreskin fibroblasts and significantly improved cell viability in a cell viability assay [20]. Moreover, the hydrogel combined with the herbal extracts was applied in an extensive wound to assess the curing ability. However, investigations of the physical-mechanical properties of hydrogels are lacking. Thus, we studied the physical chemistry properties of herbal extract-loaded hydrogels by Fourier transform infrared spectroscopy (FTIR), TGA, rheometry, and scanning electron microscopy (SEM) and analyzed the healing effect on the treatment of acne vulgaris in this research.

## 2. Materials and Methods

### 2.1. Green Tea Extract

Green tea leaves were extracted using hot water at 85–95°C, and a 5.0% solid content was achieved by applying 5 liters of hot water per kg of tea leaves. The solution was filtered using a 10-micron polypropylene filter bag and concentrated to 20% (*w*/*v*) after low-temperature vacuum drying. The extract was obtained by adding maltodextrin at a weight ratio of 2 : 1. Green tea extracts were frozen at -35°C, freeze-dried for 72 hours (0–50 hours at below 0°C and 50–72 hours increasing to 45°C), and pulverized to produce a fine tea extract powder with an average grain size of 1.0–10 *μ*m.

### 2.2. P. emblica Fruit Extract

*P. emblica* fruit extract powder was donated by Herden Technology Corporation Company (Taiwan), frozen at -35°C for 10–12 hours, and dried at 6°C for 35 hours to achieve a moisture content of less than 5.0% (*w*/*v*). The dried *P. emblica* fruit was mixed with hot water at a ratio of 1 : 5 to produce a 5.0% solid content for extraction, and then, the mixture was filtered using a 10-micron polypropylene filter bag. After concentration in a vacuum, the concentration of the extract solution was increased to 10% *w*/*v*. An additional extract of maltodextrin at a ratio of 1 : 1 (10% *P. emblica* content, 10% maltodextrin; *w*/*v*) was blended. *P. emblica* condensate was frozen at -35°C, freeze-dried for another 72 hours (0–50 hours at 0°C and 50–72 hours at 45°C), and ground to a powder for dissolution into dimethyl sulfoxide. Dulbecco's modified Eagle's medium at suitable doses (0.05, 0.1, 0.25, 0.5, and 1 mg/mL) was used to dilute the cells for the subsequent experiments.

### 2.3. Ginger (Z. officinale Rosc.) Extract

Ginger or ginger root is widely used as a flavor spice and folk medicine because it has been reported to have potential anti-inflammatory, antioxidative, antithrombosis, and possible antiallergic effects in previous papers [6, 8]. Experimental ginger material was purchased from Shennong Valley Farm Co. (Taiwan), washed and sterilized, and cut into thin slices. The slices were dried in a low-temperature environment until they were easily broken and then filtered through a 40-mesh screen for sterilization using ozone.

### 2.4. Preparation of Herbal Ingredients

The three extracts underwent extraction using water and alcohol. All natural herbal extracts in powder form (green tea, ginger, and *P. emblica* fruit) and salicylic acid were mixed in specific proportions, and water and ethanol were added. The mixtures were stirred at 95°C for 24 hours, and the extract was allowed to settle for 12 hours for further stratification. The supernatant was sterilized and filtered using 0.22 *μ*m nylon filter membranes, irradiated with UV, and stored at room temperature.

### 2.5. Herbal Extract-Loaded Hydrogel Preparation

To determine the coating properties of polymer gels and adhesion at ambient temperature, a 9% (*w*/*w*) aqueous herbal extract gelatin solution and 10% (*w*/*w*) alcohol herbal extract CMC solution were prepared. A 1–10 mL aqueous sterile water extract was added to 0.09–0.9 g gelatin and stirred to dissolve at 100°C to produce a 9% (*w*/*w*) herbal gelatin colloidal solution. The 9% (*w*/*w*) gelatin solution and the 10% (*w*/*w*) CMC solution without herbal extracts at a ratio of 10 : 1 were compared with other hydrogels. Distilled water and ethanol were used as solvents to form nonherbal hydrogels. The herbal extract-loaded hydrogels were mixed at various ratios with gelatin/CMC solution, gelatin was dissolved in aqueous herbal extract, and CMC was dissolved in alcoholic herbal extract. Herbal hydrogels with different ratios of gelatin-herbal water extract/CMC-herbal alcohol extract were named nonherbal hydrogels, hydrogels (10 : 1), hydrogels (1 : 1), and hydrogels (1 : 10). The freeze-dried hydrogel was prepared with a freeze dryer (FDU-1200, Korea) and observed using a microscope and SEM. The dried hydrogel patches were comminuted to fine powder to allow FTIR analysis and thermal gravimetric analysis (TGA).

### 2.6. FTIR

FTIR (PerkinElmer Spectrum 100) was applied from 4000 to 450 cm^−1^ at room temperature to obtain information about the biochemical compositions of the herbal extract-loaded hydrogels. The FTIR spectra of pure CMC, pure gelatin, nonherbal hydrogel, hydrogel (10 : 1), hydrogel (1 : 1), and hydrogel (1 : 10) were used to identify different functional groups. The interactions between CMC, gelatin, and herbal extracts were determined by the spectral signals.

### 2.7. TGA

TGA was performed using a PerkinElmer DMA 7e dynamic mechanical analyzer to determine the storage modulus of our biocompatible herbal extract-loaded hydrogels. Small dried powder quantities (0.5 to 5 mg) were wrapped and placed in a platinum pan. All samples were heated from 30 to 700°C at a heating rate of 10°C/min in an isolated environment that was purged of air. The TGA data were plotted as temperature versus weight (%) and heat flow (mW/mg) to verify the thermal behaviors of the hydrogels.

### 2.8. Rheology Properties

The mechanical properties of hydrogels with different ratios of CMC and gelatin were evaluated on a rheometer. The reaction solutions of hydrogels were mixed in a syringe and quickly placed on the plate of a Brookfield DV-III Ultra rheometer (Brookfield Asset Management Inc, Canada). The sample was placed in the cylindrical vessel and allowed to equilibrate at a specific temperature. To control the temperature, the water jacket of the stainless steel cylindrical vessel was connected to a constant temperature bath. The temperature was maintained at 25 ± 0.1°C. The viscosity reading was taken after 60 s for each sample [21–23]. Rheological measurements of the polymer-based nonherbal hydrogel and herbal hydrogel (10 : 1) were performed using a rheometer and three different methods. (1) A strain sweep test was used to obtain the critical strain; (2) a temperature sweep test was used to determine the storage modulus of the hydrogels; and (3) a shear rate sweep test was used to determine the viscosity of the herbal extract-loaded hydrogels [24]. The strain sweep test measured the storage modulus of the hydrogel samples for a strain from 0.1 to 100%. The temperature sweep test was performed at 0.5% strain, and the temperature was increased from room temperature to 50°C at 1 rad/s. A shear rate sweep test with a shear rate of 1/s from 0.1 to 100 at 25°C was used to measure the viscosity of the nonherbal hydrogel and hydrogel (10 : 1) samples.

### 2.9. SEM

Scanning electron microscopy was applied to determine the surface morphology. Sample preparation for SEM images was performed according to previous papers [3]. The hydrogels were allowed to reach swelling equilibrium and cut into small pieces to expose the inner surface and then pretreated with freeze-drying to completely remove the water. Freeze-dried hydrogel (10 : 1) samples were analyzed using a TOPCON scanning electron microscope (ABT-150S, Tokyo, Japan) at an acceleration voltage of 15 kV. Dried patches were sectioned using a scalpel, and cross-sections were coated with platinum and then placed on an optimally sized aluminum stub using double-sided tape. The gold coating of 300 A on the stub was deposited using gold sputtering. The structural morphology of hydrogel patches was scanned, and photomicrographs were captured.

### 2.10. Determination of 1,1-Diphenyl-2-picrylhydrazyl (DPPH) Scavenging Activity

The mechanism for DPPH scavenging activity involved the DPPH reagent accepting an electron or hydrogen radical to become a stable molecule to detect oxidative activity. Hydrogen was produced when DPPH reacted with antioxidant agents. The amount of DPPH and its absorbance decreased, and the absorbance was measured spectrophotometrically at 517 nm [10]. Vitamin C (100 *μ*M) was used as a positive control due to its superior antioxidant properties. One microliter of different concentrations of herbal polymer-based hydrogel was added to 99 *μ*L DPPH solution (0.1 mg/mL). Various sample amounts were dissolved in methanol for each well to yield a final working volume of 100 *μ*L. Distilled water was used as the control. Absorbance was measured using a spectrophotometer, and the DPPH value was plotted for comparison with the initial concentration of DPPH to determine the reduction by the antioxidant. The scavenging capacity (%) was calculated as

(1) $$\begin{array}{c} \text{Scavenging ability }\left(\%\right)=\left[1‐\left(\frac{A_{\text{Sample}}}{A_{\text{Blank}}}\right)\right]\times 100\%. \end{array}$$

### 2.11. Metal Chelating Ability

The chelation of ferrous ions in hydrogel samples was measured using a procedure developed in previous studies [25]. A 10 *μ*L aliquot of 2 mM FeCl_2_·4H_2_O solution was mixed with 1 *μ*L of different concentrations (0.5–50 mg/mL) of samples. When the sample solution was added to 20 *μ*L of 5 mM ferrozine, the complexes of ferrous ions and ferrozine changed color. A lower absorbance signified better metal chelating activity. The absorbance was measured spectrophotometrically at 562 nm. Distilled water was used as the control. A 100 *μ*M EDTA solution was used as a positive control. The chelating ability was calculated using a formula similar to Equation (1).

### 2.12. Case Report

For the clinical trials, twenty-four clinical subjects were treated with the herbal polymer-based hydrogel for a period of two weeks by a dermatologist. The Institutional Review Board proof is shown in Figure S1 ( M2019011). All individuals who participated in this study provided written informed consent to publish these details (Table S1). All skin conditions were recorded before and after the treatments, and the recovery area for inflamed acne wounds was quantified using ImageJ software (National Institutes of Health, Bethesda, USA). The results are shown in Table S1. The wound healing rate (%) was calculated using

(2) $$\begin{array}{c} \text{Wound healing rate }\left(\%\right)=\frac{W_{\text{b}}-W_{\text{a}}}{W_{\text{b}}}\times 100\%, \end{array}$$

where *W*_b_ is the wound area before treatment and *W*_a_ is the wound area after treatment.

### 2.13. Statistical Analysis

Differences between the *in vivo* experiments for the vehicle control group and the herbal polymer-based gel group were analyzed using Student's *t*-test. One-way ANOVA was used for statistical comparisons between the vehicle control group and experimental groups. A significant difference (∗) was defined as *p* < 0.05.

## 3. Results

### 3.1. Hydrogel Composition

To determine the optimal blending ratio for CMC solution with alcohol herbal extract and gelatin solution with aqueous herbal extract, the configurations of hydrogels with various mixing ratios of two types of solutions were measured. We named the nonherbal hydrogel (gelatin/CMC only) and the hydrogels with gelatin/CMC: herbal extract ratios of 10 : 1, 1 : 1, and 1 : 10 nonherbal hydrogel, hydrogel (10 : 1), hydrogel (1 : 1), and hydrogel (1 : 10), respectively. The solution formed an herbal extract-loaded hydrogel, and the solvent in the nonherbal hydrogel was substituted with dilute water and 95% ethanol. Figure S2(A-D) presents the results for the alcohol herbal extract CMC solution, the aqueous herbal extract gelatin solution, and the final hydrogel. During the gelatin process, the hydrogel became stratified, and the precipitate was deposited in the bottle of the tube as the volume of the gelatin solution increased. Figure S2(D) shows that the herbal hydrogel with a blend ratio of 1 : 10 was separated into two layers, and the precipitate was insoluble. Incomplete formation of hydrogel (1 : 10) was noted. Figure S2(E) shows freeze-dried samples with various blend ratios.

### 3.2. Hydrogel Evaluation by Structural, Thermal, and Morphological Analyses

#### 3.2.1. FTIR Analysis

**Figure 1:**
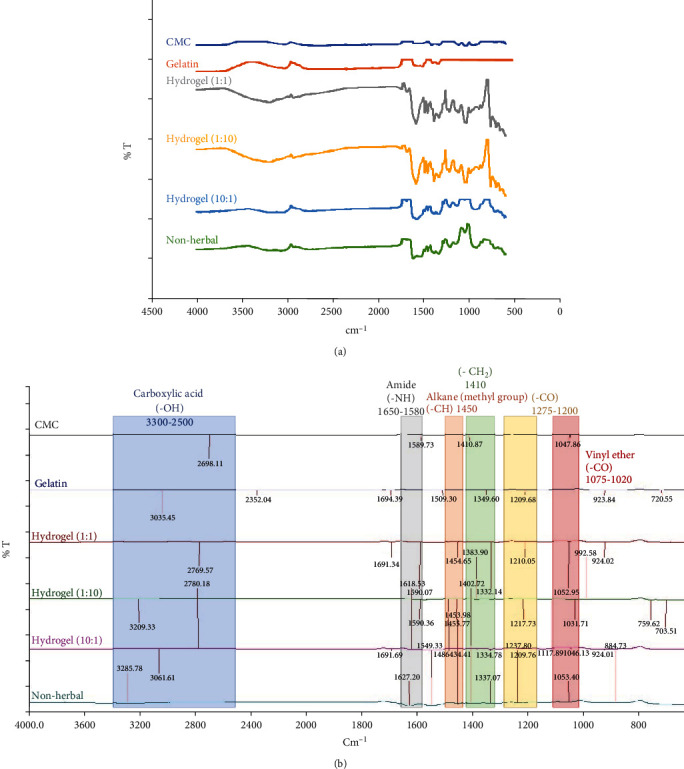
(a) The FTIR data and (b) the characteristic peaks for pure CMC, gelatin, and herbal extract-loaded hydrogels at various mixing ratios with or without herbal extract. The different hydrogel samples included nonherbal hydrogels, hydrogels (1 : 1), hydrogels (1 : 10), and hydrogels (10 : 1).

Figure 1 shows the FTIR spectra for pure CMC, gelatin powder, and herbal extract-loaded hydrogels with different ratios, which were used to identify the bands attributed to the functional groups. The CMC powder characteristic bands at 2698 and 1047 cm^−1^ were assigned to –OH stretching regions from carboxylic acid and –CO stretching regions from vinyl ether stretching and -C-O- in the ether group (−CH-O−CH_2_), respectively. The characteristic band at 1410 cm^−1^ corresponds to scissor-like -CH_2_ [26]. These absorption bands are in good agreement with reports from a previous paper [27]. The characteristic peaks in the FTIR spectrum for gelatin powder at 3035, 1694, and 1209 cm^−1^ were attributed to –OH, –NH, and –CO functional groups, respectively [26]. The hydrogel (1 : 1) features absorption bands at 2769, 1590, 1454, 1332, 1210, and 1052 cm^−1^ related to the carboxylic acid peak (–OH stretching), the amide peak (–NH bending), alkane from the methyl group (–CH bending), and scissor-like -CH_2_, -CO, and –CO functional groups. The absorption bands for hydrogel (1 : 10) at 3209, 1590, 1455, 1383, 1217, and 1031 cm^−1^ corresponded to –OH, –NH, –CH, -CH_2_, –CO, and –CO functional groups. The characteristic peaks for hydrogel (10 : 1) at 3061, 2780, 1618, 1454, 1334, 1209, and 1046 cm^−1^ were attributed to –OH, –OH, –NH, –CH, -CH_2_, –CO, and –CO functional groups. The nonherbal hydrogel features peaks at 3285, 1627, 1453, 1402, 1337, 1237, and 1053 cm^−1^ attributed to –OH, –NH, –CH, -OH, -CH_2_, –CO, and –CO functional groups.

The FTIR spectra of gelatin, CMC, and herbal extract-loaded hydrogels showed that the –CH bending band for the alkane in herbal hydrogels with various ratios at approximately 1450 cm^−1^ was not present in the IR spectra for pure CMC or gelatin powder because the interactions between gelatin and CMC formed a new linkage after cross-linking. The -CH_2_ functional group in CMC was shifted to approximately 1335 cm^−1^, demonstrating that CMC and gelatin were integrated into polymer-based hydrogels and caused conformational changes. According to a previous paper [28], the band at approximately 1651 cm^−1^ attributed to CMC provided information on the helicity of the protein in herbal extract-loaded hydrogels. A broad absorption band at 3300 cm^−1^ for OH groups corresponded to absorbed water, secondary alcohols (CMC), and (intramolecular/intermolecular) hydrogen bonding. The amide І band corresponding to –CO stretching vibration was the best region in the spectrum to show changes in the secondary structure of the protein. In the composite film, the amide I peak shifted from 1589 cm^−1^ to approximately 1615 cm^−1^. The peaks between 1209 and 1694 cm^−1^ were likely due to CN stretching and NH in-plane deformation vibrations from peptide groups in the amide I, amide II, and amide II moieties of gelatin. The absorption bands for different herbal hydrogels at approximately 3000, 1590, 1450, 1350, 1210, and 1050 corresponded to -OH, -NH, -CH, -OH, -CO, and -CO functional groups in CMC and gelatin, respectively. Therefore, the incorporation of CMC and gelatin into herbal extract-loaded hydrogels caused chemical conformational changes.

#### 3.2.2. TGA

**Figure 2:**
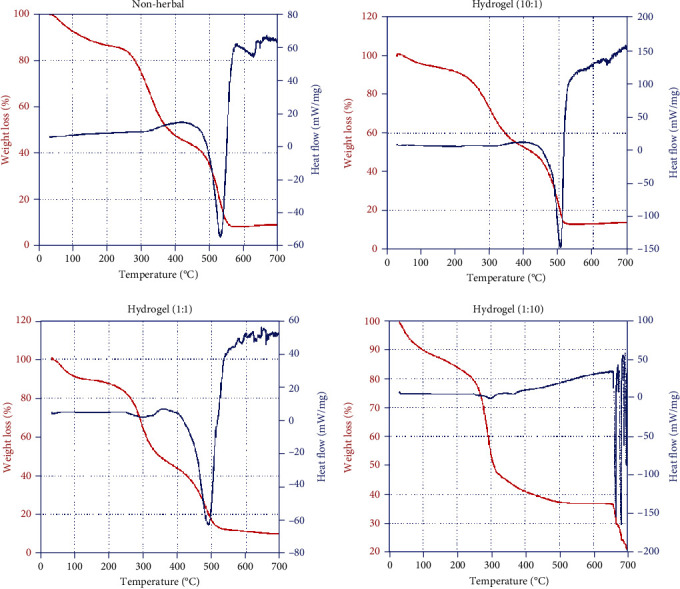
TGA results for the nonherbal hydrogel, hydrogel (1 : 1), hydrogel (1 : 10), and hydrogel (10 : 1) samples. The thermal properties of the samples were determined using a thermal analyzer to determine weight loss and heat flow.

TGA in this research helped determine the stability of the biocompatible herbal extract-loaded hydrogel, and the results are shown in Figure 2. All hydrogels underwent two or three major degradation steps, as presented by the TGA curves. Thermal degradation, including water loss, combustion, and hydrogel decomposition, was observed for all samples. No additional thermal event was noted for native and functionalized hydrogels; thus, no impurities or byproducts were observed. Initial weight losses of 14.78%, 12.11%, 15.98%, and 19.44% were observed for the nonherbal hydrogel, hydrogel (10 : 1), hydrogel (1 : 1), and hydrogel (1 : 10), respectively, until 240°C. The decrease in weight was due to the elimination of the moisture retained by the materials and the evaporation of adsorbed water. The significant reduction in moisture content was a result of a strong hydrogen bonding interaction [29]. Weight loss was found to be less than 20% in the hydrogel at 200°C. The weight losses in pure carboxymethyl cellulose (CMC) and pure gelatin were approximately 130°C and 100°C, respectively, suggesting that the hydrogel composed of gelatin, CMC, and herbal extract helped stabilize the hydrogel structure [30, 31].

Strong stretching loss occurred in the nonherbal hydrogel, hydrogel (10 : 1), and hydrogel (1 : 1) between 240 and 380°C, and the values for weight loss were 49.79%, 45.45%, and 53.90%, respectively. Weight loss measurements began at approximately 240°C and ended at approximately 380°C, mainly due to depolymerization of the carbohydrate polymer [26, 29]. The weight loss from 240°C to 380°C was due to the remaining fraction of volatile compounds of herbal extract and nondegraded CMC side branches [30]. At a temperature of less than 700°C, the retained weights of the hydrogel were 9.12%, 13.86%, 10.20%, and 20.78% for nonherbal hydrogel, hydrogel (10 : 1), hydrogel (1 : 1), and hydrogel (1 : 10), respectively. The weight loss at higher temperatures (approximately 700°C) was sluggish, resulting in no separate or major phase in the composite ingredients pertaining to a specific TGA behavior [30] and thus indicating that some organic materials (CMC and gelatin) were still present in the herbal extract-loaded hydrogels [32].

Two-stage weight loss was observed for the nonherbal hydrogel, hydrogel (10 : 1), and hydrogel (1 : 1), which behaved similarly to the three hydrogels with different blend ratios. Weight loss was slower for the hydrogel containing more gelatin during the first stage of weight loss (to 240°C). Weight loss in hydrogel (1 : 10) decreased faster than that in the other hydrogels at both stages. A volatile decrease in weight loss was observed for hydrogel (1 : 10) from 240 to 600°C. This behavior was different for other hydrogels, possibly because the structure of hydrogel (1 : 10) was incomplete and the components of CMC and gelatin were not mixed well for the various ratios of the hydrogels. The results were similar to the TGA data for pure CMC or pure gelatin (data not shown), which showed incomplete cross-linking between the CMC and gelatin.

Each thermogram exhibited a single exothermic peak at 526°C, 504°C, and 489°C for the nonherbal hydrogel, hydrogel (10 : 1), and hydrogel (1 : 1), respectively. However, no significant exothermic peak was observed in the TGA data for hydrogel (1 : 10), possibly because of the incomplete synthesis of gelatin and CMC at the blend ratio of 10 : 1. More specifically, the addition of CMC to gelatin decreased the melting point of the hydrogel. The interaction between the two macromolecules increased, and CMC plasticized the gelatin for the nonherbal hydrogel and hydrogel (10 : 1). The cross-linked hydrogel was more stable at a blend ratio of 10 : 1 [28]. For both blended gelatin/CMC hydrogels, the glass transition values showed that the cross-linking reaction caused by the addition of CMC affects the thermal properties of the hydrogel. Crosslinking of the gelatin macromolecule and CMC increased the thermal stability of the gelatin hydrogel [33].

#### 3.2.3. Rheological Analysis

**Figure 3:**
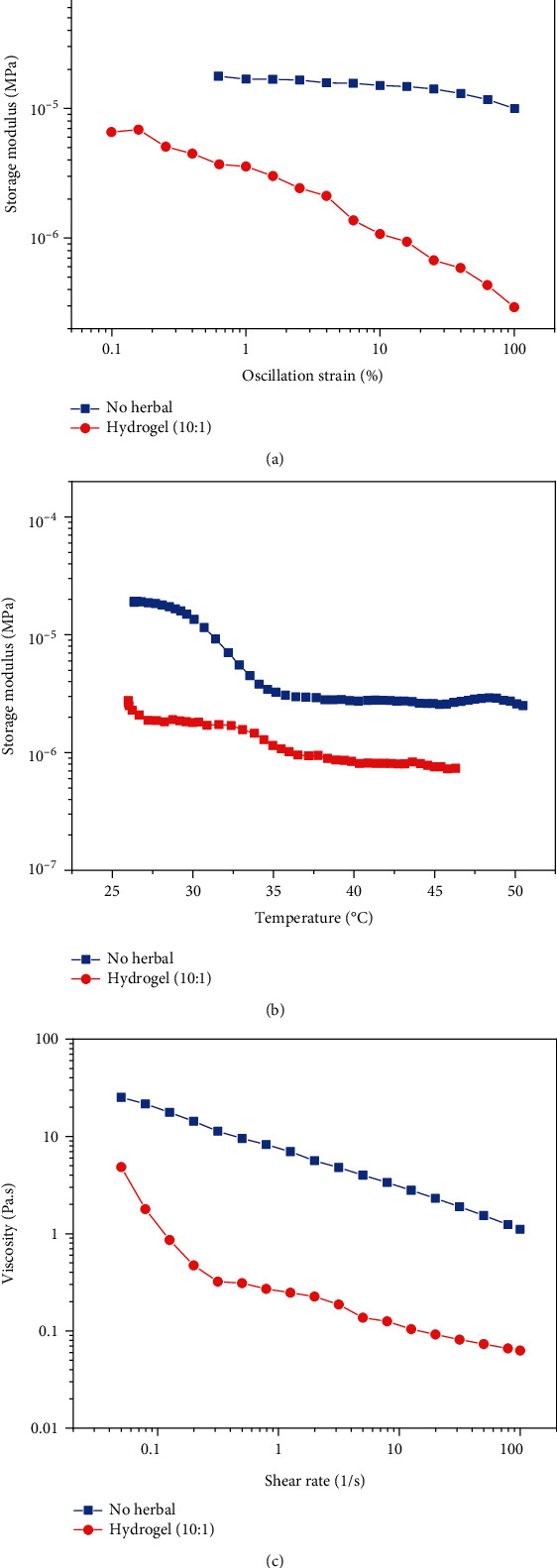
(a) The rheological properties of the storage modulus for a strain sweep ranging from 0.1 to 100% for the nonherbal hydrogel and hydrogel (10 : 1). (b) The relationship between temperature and the storage modulus for the nonherbal hydrogel and hydrogel (10 : 1) at 25°C. (c) The viscosity of the nonherbal hydrogel and hydrogel (10 : 1). The shear-thinning behavior of herbal extract-loaded hydrogels was characterized by an increasing shear rate from 0.1 to 100 (1/s).

The rheological behaviors of the herbal extract-loaded hydrogels were characterized to determine the wound healing properties using a rheometer. The storage modulus was measured using an oscillating strain at a fixed angular frequency (1 rad/s). When the angular frequency changed from 0.1 to 100 strains (%), the hydrogel storage modulus presented no significant change between 0.1 and 10 strains; thus, the hydrogels were stable (Figure 3(a)) [23]. The variation in the storage modulus due to temperature for herbal extract-loaded hydrogels with a blend ratio of 10 : 1 is shown in Figure 3(b). Both hydrogels behaved similarly in that the storage modulus decreased as the temperature increased. Measurement of viscosity as a function of the shear rate showed that the viscosity of the nonherbal hydrogel and hydrogel (10 : 1) decreased as the shear rate increased (Figure 3(c)), which is known as shear-thinning behavior [34]. Shear-thinning behavior resulting in high viscosity at low shear rates has been observed in biological fluids such as biomaterials or gelling products. To increase firmness, stability, and durability, shear-thinning subjects are needed. The shear rate increased as viscosity decreased, and products that exhibited shear-thinning behavior were therefore absorbed easily onto the skin through topical application. Herbal extract-loaded hydrogels are an ideal synthetic film for wound repair applications because they remain on the skin [34].

### 3.3. Morphology and Antioxidant Ability of Herbal Hydrogels

#### 3.3.1. Morphological Analysis

**Figure 4:**
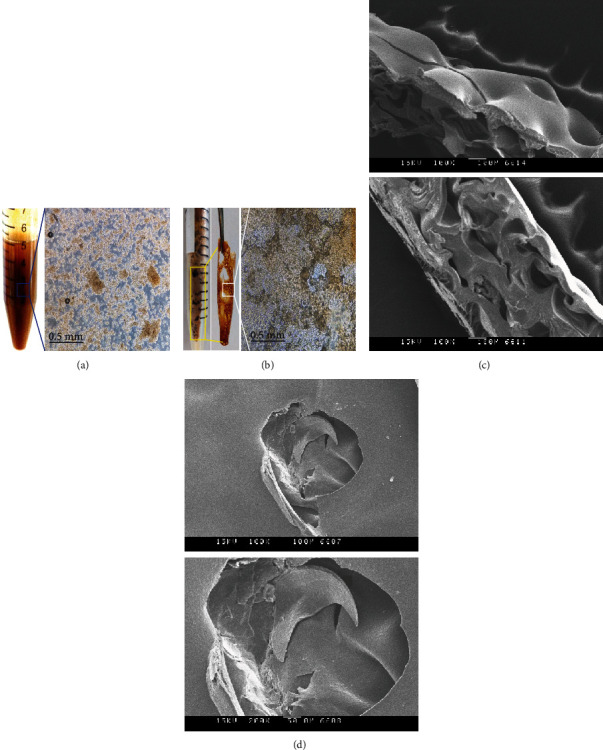
The morphology of the hydrogel (10 : 1), which comprised 9% (*w*/*w*) herbal gelatin mixed with 10% (*w*/*w*) alcohol herbal extract CMC solution: (a) microscopic images of the hydrogel and (b) the freeze-dried sample. The morphology of the hydrogel (10 : 1): (c) the cross-sectional interface and (d) the surface structure scanned by SEM.

Herbal water extract with a specific ratio of herbal alcohol extract was stirred at room temperature until it completely dissolved. Both solutions at a specific ratio were dispensed to form an herbal polymer-based gel. Alcohol extraction increased the extraction rate and allowed quick drying of the resultant gel. Figures 4(a) and 4(b) show microscopic images of the morphological features of the CMC/gelatin/herbal extract hydrogel. The microstructure was an interlaced, highly concatenated porous structure, and the surface with multiple apertures was tightly connected after freeze-drying. Figures 4(c) and 4(d) show the SEM images of the nonherbal hydrogel and hydrogel (10 : 1). These SEM images showed that the lyophilized hydrogels were porous and feature an interconnected polymer network with a multilayered structure. Live cells could be incorporated into the hydrogel and stored in multiple layers [35].

#### 3.3.2. Antioxidant Activity

**Figure 5:**
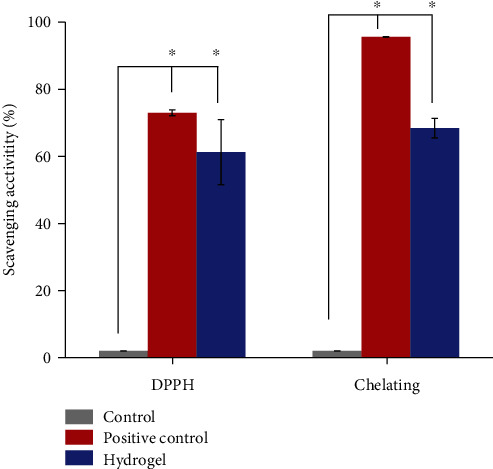
The antioxidant ability in DPPH and the chelating scavenging activity of herbal extract-loaded hydrogels.

DPPH scavenging capability is a vital element for functional foods and skin care because excessive accumulation of free radicals accelerates the oxidation of lipids in foods and cosmetics [8]. During the reaction for DPPH free radical scavenging, antioxidants inhibit oxidation products. The scavenging value for the DPPH radical was used to determine antioxidant activity. Ferrozine forms complexes with Fe^2+^ quantitatively. The construction of the complex was disrupted by the presence of chelating agents, and the red color of the complex faded.  Figure 5 shows the DPPH scavenging activity and metal chelating ability of the herbal extract-loaded hydrogel used in this study. The results showed that the herbal hydrogels increased DPPH scavenging activity and chelating ability by 61.25 ± 7.07% and 83.93 ± 9.68%, respectively. This figure shows that DPPH scavenging activity was significantly higher in the experimental groups than in the control group. Antioxidants convert the stable radical DPPH into yellow-colored diphenyl-picrylhydrazine. The herbal hydrogel inhibited DPPH more than vitamin C. The chelating value for the herbal extract-loaded hydrogel produced using herbal extract showed a small Fe^2+^ scavenging value because EDTA solution exhibits a moderate-high scavenging capability.

### 3.4. Case Report

**Figure 6:**
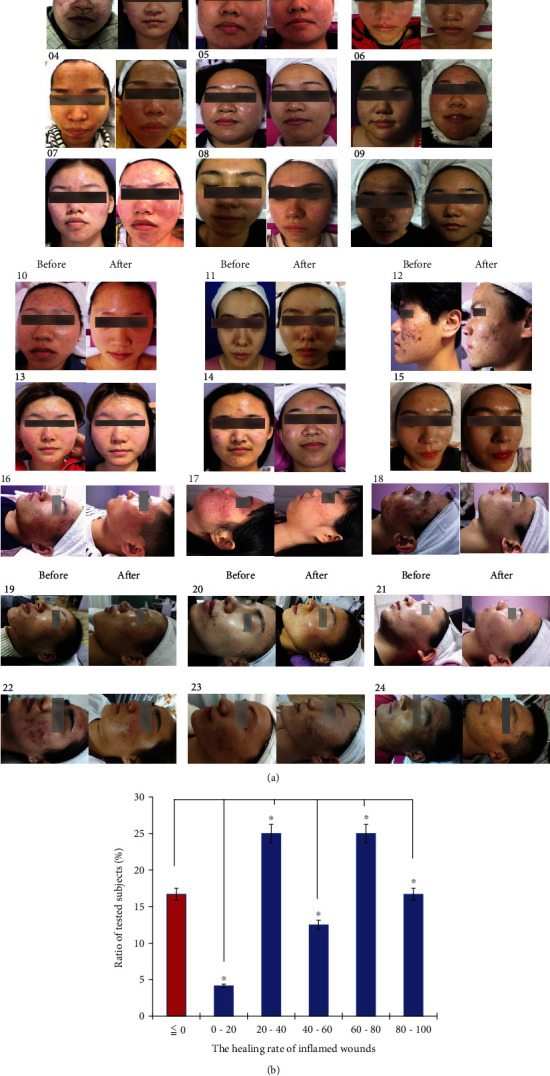
(a) Images of twenty-four clinical subjects treated with the herbal extract-loaded hydrogel at the beginning and end of 14 days of treatment and (b) the quantitative healing rate for acne with the gel treatments.

The patients with moderate to severe acne volunteered to accept the whole course of treatment with herbal extract-loaded hydrogels. They were instructed to use the hydrogel to cover acneic areas after cleaning their faces three times per day and to consistently apply this moist wound dressing for 14 days. The results of the human trials are shown in Figure 6. A total of 16.7% of twenty-four individuals showed a recovery area greater than 80%, and 34.28% of the patients experienced a wound healing rate of more than 75%. Seventy-five percent of participants had a repair ratio greater than 30%, and 20.83% of individuals had a recovery ratio of less than 20%. Approximately 54% of the patients had a healing rate greater than 40%. No uncontrollable factors for clinical trials involving human subjects were identified. The volunteers remained at normal temperature and humidity for 14 days, and their daily routine, diet, and intake of liquids were fixed due to their general lifestyles. Some clinical subjects (16.7%) had a specific physiological status or living habits that affected wound healing. The results show that the herbal extract-loaded hydrogel had a positive effect on most clinical subjects, and the biomaterial was effective for curing acne.

## 4. Discussion

More than 90% of Australian adolescents aged 16–18 years suffer from acne vulgaris, and their self-esteem and emotional state can be affected. Moderate to severe acne can lead to scarring [36]. Many causes of acne vulgaris have been identified, including stress, irregular sleeping habits, lack of sleep, a high-calorie or high-sugar diet, hormonal changes, acne position comparison, improper use of skin care products or cosmetics, a dirty environment, and certain oral medications. Acne is caused by the action of bacteria called *Propionibacterium acnes*, keratinization of follicular keratinocytes, sebum production from sebaceous glands, and the inflammatory response of skin. Acne vulgaris can cause more serious problems, including boils, cellulitis, scars, and keloids, due to bacterial infections and sequelae if treated improperly. The etiology is unclear and probably involves multiple factors; therefore, targeted and low-risk treatments have yet to be developed [36]. Pharmacological therapies, including isotretinoin, benzoyl peroxide, and antibiotics, are often used to treat acne in adolescents [1]. These types of acne medications act by reducing oil production and swelling or by inhibiting bacterial infection. Many months may be necessary to cure acne completely, and some agents heal acne quickly.

This study developed an herbal extract-loaded hydrogel containing green tea extract, ginger, *P. emblica*, and salicylic acid to cure moderate to severe acne completely in two weeks. More than half of the twenty-four test subjects had a 50% healing rate. Current prescription drugs may not produce a change for four to eight weeks, but the herbal extract-loaded hydrogel cured acne rapidly, possibly due to the active ingredients and protection of the skin barrier from outer pathogens. Anti-inflammatory, anti-irritant, and antibacterial effects are the pharmacological features of natural products such as green tea, *P. emblica*, and ginger extracts and accelerate the wound healing process. Tea polyphenols have been shown to reduce the severity of acne, and green tea extract significantly reduced the number of forehead, cheek, and total lesions after 4 weeks of treatment [4]. The anti-inflammatory and keratolytic effects of salicylic acid have been studied [1]. Salicylates with anti-inflammatory and postinflammatory properties reverse the hypercornification of the follicular canal and facilitate the expulsion of existing comedones by inducing accelerated proliferation of the follicular epithelium [22]. The wound healing effect of gel with natural extracts on full-thickness wounds was reported in one study [20]. This hydrogel achieved water-based transmission to release the active components from the hydrogel. The functional agents achieved the astringent effect of tightening the skin and inhibiting sweat and sebum secretion to temporarily shrink keratin without the problem of drug resistance and sensitization. To enter the cosmetic market, comparative studies with established therapies and combination therapies are necessary. The herbal extract-loaded hydrogel efficiently healed moderate to severe acne. The preliminary clinical trial demonstrated the hydrogel's efficacy in healing acne rapidly and reducing epithelialization.

## 5. Conclusions

This study mixes herbal extracts, including green tea, *Z. officinale* Rosc., *P. emblica* ingredients, and salicylic acid, with gelatin and CMC to develop an herbal polymer-based hydrogel. The resultant gel was used in an *in vivo* experiment to determine its efficacy in controlling sebum secretion and its anti-inflammatory effect. Polymer-based hydrogels with good mechanical properties, rheological characteristics, and surface morphologies were retained around injured skin, and the microstructure of the gel allowed cells to reside within to support wound repair. The results showed that the addition of herbal extract to the gelatin/CMC gel reduced inflammation and improved the rate of acne closure after 14 days of treatment. More than fifty percent of the twenty-four test subjects showed 50% recovery from moderate-to-severe acne problems. Further study is required to quantify the inflammatory cells and the reduction in sebum production. This preliminary study showed that the proposed hydrogel can heal acne rapidly and reduce epithelialization.

## Data Availability

The data that support the findings of this study are available from all authors upon reasonable request.

## Supplementary Materials

Supplementary Materials Figure S1: IRB certificate for human examination ( M2019011). Figure S2: images of herbal extract-loaded hydrogels at various mixing ratios of gelatin herbal extract and CMC herbal extract. Table S1: the wound healing areas of the 24 subjects.
