# Supplementary material for: A Novel Biocompatible Herbal Extract-Loaded Hydrogel for Acne Treatment and Repair
Source: Oxid Med Cell Longev. 2021 Nov 2;2021:5598291. doi: 10.1155/2021/5598291 (PMC8577930; doi:10.1155/2021/5598291)
Supplement: Supplementary Materials — Figure S1: IRB certificate for human examination ( M2019011). Figure S2: images of herbal extract-loaded hydrogels at various mixing ratios of gelatin herbal extract and CMC herbal extract. Table S1: the wound healing areas of the 24 subjects. [file 5598291.f1.docx]

Supporting information

A novel biocompatible herbal extract-loaded hydrogel for acne treatment and repair

Ying-Yi Lin^1,†^, Shu-Hsu Lu^2,†^, Rong Gao^3^, Chia-Hung Kuo^4^, Ching-Chou Wu^5,6^, Wei-Chih Lien^7^, Wen-Hisn Chung^8^, Yong Diao^9,^*, Hui-Min David Wang ^1,6,10,11,^*

^1^ Graduate Institute of Biomedical Engineering, National Chung Hsing University, Taichung City, Taiwan.; [a0979169597@gmail.com](mailto:a0979169597@gmail.com)

^2^ Division of Cardiology, Department of Internal Medicine, Kaohsiung Armed Forces General Hospital, Kaohsiung City, Taiwan.; [Lucats0709@gmail.com](mailto:Lucats0709@gmail.com)

^3^ Deloitte Institute of Biology, Yangtze River Delta Research Institute, Tsinghua University, Beijing, China.; [gaorong709@naver.com](mailto:gaorong709@naver.com)

^4^ Department of Seafood Science, National Kaohsiung University of Science, Kaohsiung City, Taiwan.; [kuoch@nkust.edu.tw](mailto:kuoch@nkust.edu.tw)

^5^ Department of Bio-Industrial Mechatronics Engineering, National Chung Hsing University, Taichung City, Taiwan.; [ccwu@dragon.nchu.edu.tw](mailto:ccwu@dragon.nchu.edu.tw)

^6^ Ph.D. Program in Tissue Engineering and Regenerative Medicine, National Chung Hsing University, Taichung City, Taiwan.;

^7^ Department of Physical Medicine and Rehabilitation, National Cheng Kung University Hospital, College of Medicine, National Cheng Kung University, Tainan, Taiwan.; [lwclwhab@ms8.hinet.net](mailto:lwclwhab@ms8.hinet.net)

^8^ Department of Plant Pathology, National Chung Hsing University, Taichung City, Taiwan.; [wenchung@nchu.edu.tw](mailto:wenchung@nchu.edu.tw)

^9^ School of Medicine, Huaqiao University, Quanzhou, Fujian Province, China.;

^10^ Graduate Institute of Medicine, College of Medicine, Kaohsiung Medical University, Kaohsiung City, Taiwan.;

^11^ Department of Medical Laboratory Science and Biotechnology, China Medical University, Taichung City, Taiwan.;


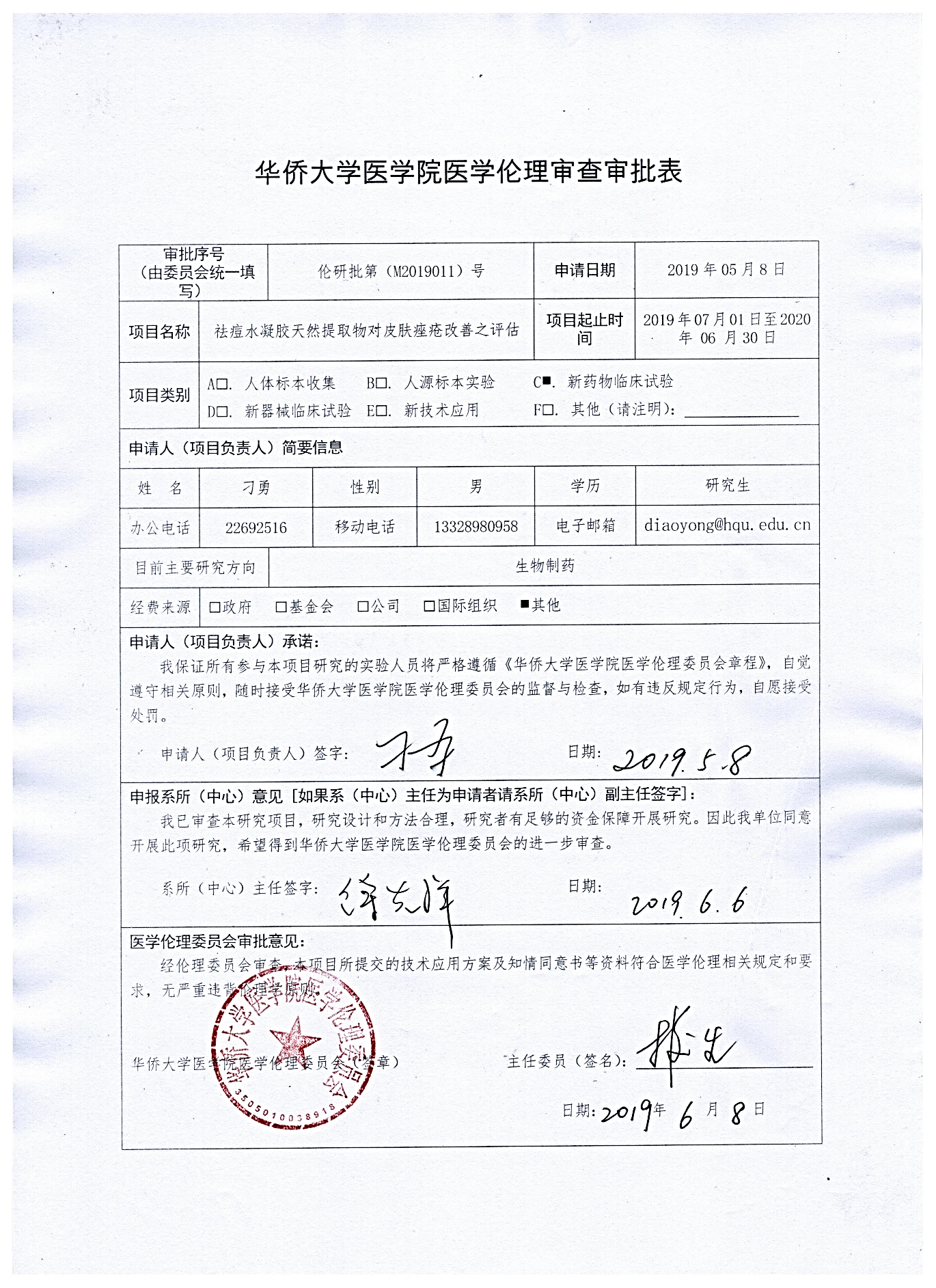
 **Figure S1**. The approval of the clinical experiment of herbal polymer-based gel.

**
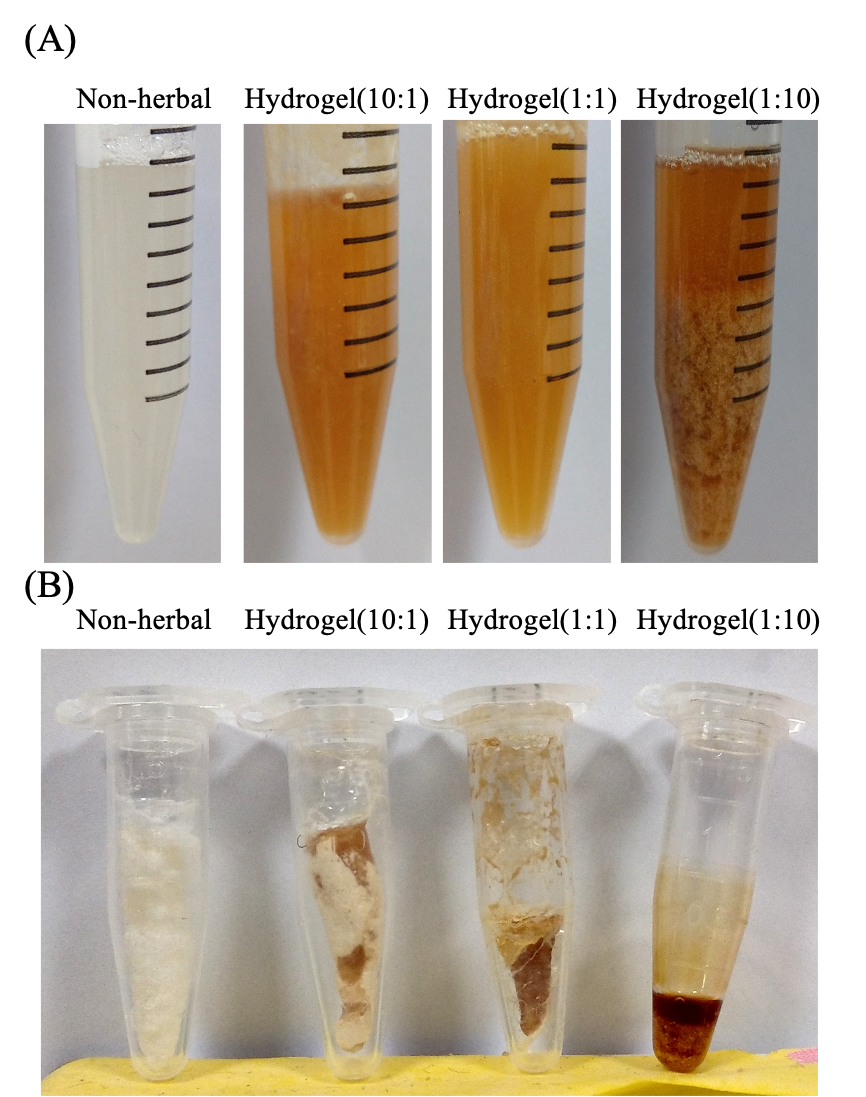
**

**Figure S2.** The images of polymer-based hydrogels in various mixing ratios with gelatin/CMC solution, and the gelatin was dissolved by aqueous herbal extract and CMC was dissolved by alcoholic herbal extract. (A) The photos from left to right were non-herbal hydrogel (gelatin/CMC only); hydrogel gelatin/CMC:herbal extract = 10:1; 1:1; 1:10, respectively; and (B) group photo of four freeze-dying hydrogels. **Table S1.** The wound healing area of 24 subjects. (Calculated by ImageJ software from National Institute of Health, Bethesda, USA)

| **No.** | **Before (pixel)** | **After (pixel)** | **Wound healing rate (%)** |
| --- | --- | --- | --- |
| 1 | 76964 | 14717 | 80.88 ± 4.04 |
| 2 | 51838 | 56065 | -8.15 ± 0.41 |
| 3 | 75401 | 21552 | 71.42 ± 3.57 |
| 4 | 110141 | 31229 | 71.65 ± 3.58 |
| 5 | 31920 | 5536 | 82.66 ± 4.13 |
| 6 | 61710 | 65301 | -5.82 ± 0.29 |
| 7 | 62460 | 42390 | 32.13 ± 1.61 |
| 8 | 63413 | 27702 | 56.31 ± 2.82 |
| 9 | 47823 | 15974 | 66.60 ± 3.33 |
| 10 | 29569 | 7109 | 75.96 ± 3.80 |
| 11 | 40561 | 77801 | -91.81 ± 4.59 |
| 12 | 41082 | 49808 | -21.24 ± 1.06 |
| 13 | 35836 | 19581 | 45.36 ± 2.27 |
| 14 | 9400 | 5721 | 39.14 ± 1.96 |
| 15 | 115775 | 68175 | 41.11 ± 2.06 |
| 16 | 173345 | 20092 | 88.41 ± 4.42 |
| 17 | 24470 | 7377 | 69.85 ± 3.49 |
| 18 | 206948 | 24049 | 88.38 ± 4.42 |
| 19 | 76526 | 56823 | 25.75 ± 1.29 |
| 20 | 92494 | 57702 | 37.62 ± 1.88 |
| 21 | 33869 | 22701 | 32.97 ± 1.65 |
| 22 | 34275 | 29587 | 13.68 ± 0.68 |
| 23 | 182669 | 70475 | 61.42 ± 3.07 |
| 24 | 79514 | 49797 | 37.37 ± 1.87 |
